# KDELR2-KIF20A axis facilitates bladder cancer growth and metastasis by enhancing Golgi-mediated secretion

**DOI:** 10.1186/s12575-022-00174-y

**Published:** 2022-09-12

**Authors:** Xiangui Meng, Weiquan Li, Hongwei Yuan, Wei Dong, Wen Xiao, Xiaoping Zhang

**Affiliations:** 1 Department of Urology, Union Hospital, Tongji Medical College, Huazhong University of Science and Technology, Wuhan, 430022 China; 2 Shenzhen Huazhong University of Science and Technology Research Institute, Shenzhen, 518000 China; 3 Institute of Urology, Tongji Medical College, Huazhong University of Science and Technology, Wuhan, 430022 China

**Keywords:** KDEL endoplasmic reticulum protein retention receptor 2 (KDELR2), Kinesin family member 20A (KIF20A), Bladder cancer (BCa), MMPs, Prognosis

## Abstract

**Background:**

Bladder cancer (BCa) is a fatal form of cancer worldwide associated with a poor prognosis. Identifying novel drivers of growth and metastasis hold therapeutic potential for the disease. Transport homeostasis between the endoplasmic reticulum and Golgi and the secretion of matrix metalloproteinases (MMPs) mediated by Golgi have been reported to be closely associated with tumor progression. However, to date, mechanistic studies remain limited.

**Results:**

Here, we identified KDELR2 as a potential risk factor with prognostic value in patients with BCa, especially those harbouring the KDELR2 amplification. In addition, we found that KDELR2 is a regulator of BCa cell proliferation and tumorigenicity based on bioinformatic analysis with functional studies. Mechanistically, we revealed that KDELR2 could regulate the expression of KIF20A, thus stimulating the expression of MMP2, MMP9 and MKI67. Functionally, the overexpression of KDELR2 and KIF20A markedly promoted proliferation, migration, and invasion in vitro and enhanced tumor growth in vivo, while knockdown of KDELR2 and KIF20A exerted the opposite effects. And the overexpression of KDELR2 also enhanced lymph node metastasis in vivo.

**Conclusions:**

Collectively, our findings clarified a hitherto unexplored mechanism of KDELR2-KIF20A axis in increasing Golgi-mediated secretion of MMPs to drive tumor progression in BCa.

**Supplementary Information:**

The online version contains supplementary material available at 10.1186/s12575-022-00174-y.

## Background

Bladder cancer (BCa) is one of the most lethal malignancies, with an increasing incidence worldwide [1]. Approximately 573,000 new cases of BCa and 213,000 deaths were reported worldwide in the past 2020 [2]. It has been estimated that 83,730 new cases of BCa will be diagnosed in the United States this year, with approximately 17,200 individuals succumbing to the disease [3]. Despite significant advances in the treatment of BCa, including traditional surgical resection, chemotherapy, radiotherapy and immunotherapy, postoperative recurrence and distant metastasis affect the five-year survival rate of patients with advanced BCa [4]. Therefore, prompt identification of the potential therapeutic targets and biomarkers for BCa is essential.

The KDEL (Lys-Asp-Glu-Leu) receptors (KDELRs), proteins with seven transmembrane domains, including KDELR1-3, localise mainly on the Golgi complex (usually cis side), the endoplasmic reticulum (ER), and the intermediate ER-Golgi compartment (ERGIC) [5]. KDELRs are known to regulate the trafficking of proteins between the Golgi and the ER, aiming to retrotransport chaperones from the Golgi complex to the ER. KDELRs play a key role in maintaining transport homeostasis between the Golgi and the ER. KDELR1-3 has different binding substrates, with KDELR2 being specific and the others being general [6]. Previous studies have shown that KDELR2 is involved in the regulation of cellular functions, including cell proliferation, survival, migration, cytoskeletal rearrangements, secretion and other biological activities [7], regulating the ER stress [8, 9], cellular secretory traffic [10], and immune responses [11]. However, the roles of KDELRs family remain unclear, especially in tumor progression. The existing research state that human glioma tissues and cell lines highly express KDELR2 [12, 13]. KDELR2 is a poor prognostic factor for glioblastoma and acts as a direct target of HIF-1α, a regulator of p-mTOR as well as the downstream protein in glioblastoma cells [12]. KDELR2 knockdown could inhibit the viability of glioma cells, promote cell cycle arrest at the G1 phase and induce apoptotic cell death by targeting CCND1 [13]. In melanoma cells, KDELR activity promoted invadopodia formation and extracellular matrix (ECM) degradation [14]. Overexpression of KDELR2 is sufficient to independently trigger lung cancer invasion and metastasis by an EMT-independent mechanism [15]. Increasing evidence has revealed that cellular KDELR2 has more complex roles in tumor progression. Nonetheless, to date, no study has demonstrated the putative role of KDELR2 and its function in the Golgi-mediated expression of proteins, as well as in the invasion and metastasis of BCa cells driven by matrix metalloproteinases (MMPs).

Comparatively, the pivotal role of the kinesin family member 20A (KIF20A) has been extensively explored in cancer. In 2015, upregulation of KIF20A was first identified in pancreatic cancer, and KIF20A inhibition appeared to significantly inhibit the proliferation of pancreatic cancer cells [16]. Subsequently, a prominent increase in KIF20A expression was observed in several malignancies, such as lung [17, 18], breast [19], gastric [20], liver [21], bladder [22], and pancreatic cancers [23, 24]. Moreover, overexpression of KIF20A was tightly associated with a poor prognosis and the clinical parameters in patients with gastric cancer [25]. Currently, although studies have reported that KIF20A induced the progression of BCa by promoting the growth, viability and metastasis of BCa cells [22], the specific regulatory and molecular mechanisms of KIF20A and its potential role in BCa tumorigenesis remains largely elusive.

Through the current study, we have provided mechanistic insights into the effect of KDELR2 activation on the behaviour of BCa cells. Our results revealed that KDELR2 positively modulates the expression of KIF20A, while knockdown of KDELR2 downregulated KIF20A and further attenuated the growth, migration, and invasion of BCa cells.

## Results

### Identification of KDELR2 upregulation is prognostically significant in patients with BCa

**Fig. 1:**
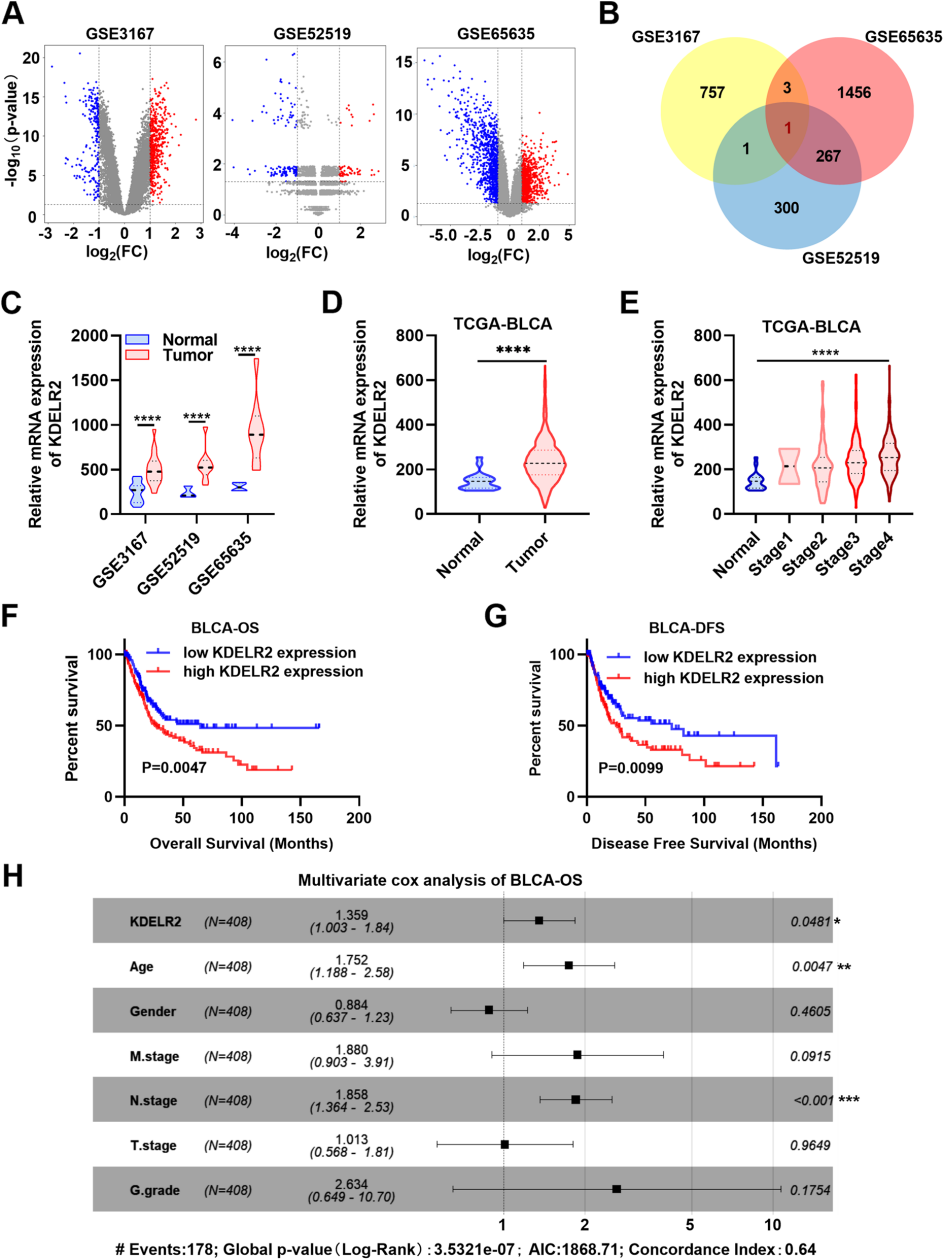
Identification and clinical characterisation of KDELR2 in BCa. **A **Volcano maps of the DEGs in BCa based on the GSE3167 (*n* = 765: upregulated, 232; downregulated, 533), GSE52519 (*n* = 573, upregulated, 236; downregulated, 337) and GSE65635 datasets (*n* = 1746, upregulated, 929; downregulated, 817). **B** A Venn diagram of the three independent GEO datasets (GSE3167, GSE52519 and GSE65635). **C** Expression profiles of KDELR2 mRNA in the three independent GEO datasets. **D** Expression profiles of KDELR2 mRNA in TCGA-BLCA dataset (normal, *n* = 19; tumor, *n* = 408). **E** KDELR2 mRNA levels at different clinical stages (normal, *n* = 19; StageI, *n* = 2; StageII, *n* = 130; Stage III, *n* = 140; Stage IV, *n* = 134). **F**-**G** Kaplan–Meier curves of KDELR2 expression in patients with BCa in TCGA-BLCA dataset (OS: high, *n* = 204; low, *n* = 204; DFS: high, *n* = 160; low, *n* = 159). **H** Multivariate analyses of the KDELR2 mRNA level and OS of patients with BCa. *p* < 0.05, *; *p* < 0.01, **; *p* < 0.001, ***; *p* < 0.0001,****

Three microarray datasets (GSE3167, GSE52519, GSE65635) were first downloaded from the GEO database to identify the candidate genes associated with the carcinogenesis and progression of BCa. The DEGs (765 in GSE3167 [upregulated, 232; downregulated, 533], 573 in GSE52519 [upregulated, 236; downregulated, 337], and 1746 in GSE65635 [upregulated, 929; downregulated, 817]) were identified (Fig. 1A). Those genes that were not differentially expressed in BCa, such as EGFR, p53, and FGFR3, were excluded (data not showed). Surprisingly, the three datasets contained one overlapping co-dysregulation gene in BCa, KDELR2, as shown in the Venn diagram (Fig. 1B). Furthermore, the results revealed that KDELR2 was significantly elevated in BCa tissues relative to normal tissues (Fig. 1C). The ROC curves, based on the GSE3167 dataset, revealed that the level of KDELR2 could differentiate BCa from normal tissues with an AUC of 0.9101 (95% CI: 0.8389 to 0.9814, P < 0.0001) (sFig. 1A). Consequently, we investigated the changes in the expression of KDELR2 in TCGA database and assessed its prognostic correlation in patients with BCa. As expected, this upregulation of KDELR2 expression was also observed in TCGA-BLCA database (Fig. 1D). Correspondingly, the in‐depth analysis revealed a significant positive correlation between the level of KDELR2 and the clinical stage of patients with BCa (Fig. 1E). The ROC curves showed that KDELR2 exhibited considerable diagnostic efficiency for BCa (*P* < 0.0001; AUC = 0.8286, 95% CI: 0.7572 to 0.8999) (sFig. 1B). Here, for the first time, Kaplan–Meier curves revealed that KDELR2 was statistically associated with the OS and DFS rates (Fig. 1F-G). The survival time of patients with BCa with low KDELR2 expression was longer than that of those with high KDELR2 expression. Similar survival analysis outcomes were observed in the additional dataset GSE32894 (sFig. 1C). Next, KDELR2 was identified through multivariate Cox regression analysis to construct a predictive signature. The multivariate analysis showed that high KDELR2 expression was an independent predictor of poor OS (HR, 1.359; 95% CI, 1.003–1.84; *p* = 0.0481) (Fig. 1H) and DFS (HR, 1.394; 95% CI, 0.993–1.96; *p* = 0.0552) (sFig. 1D). Thus, we focused on this significant prognostic or clinical outcome-related gene in our further research.

### KDELR2 functions as an oncogene in BCa cells

**Fig. 2:**
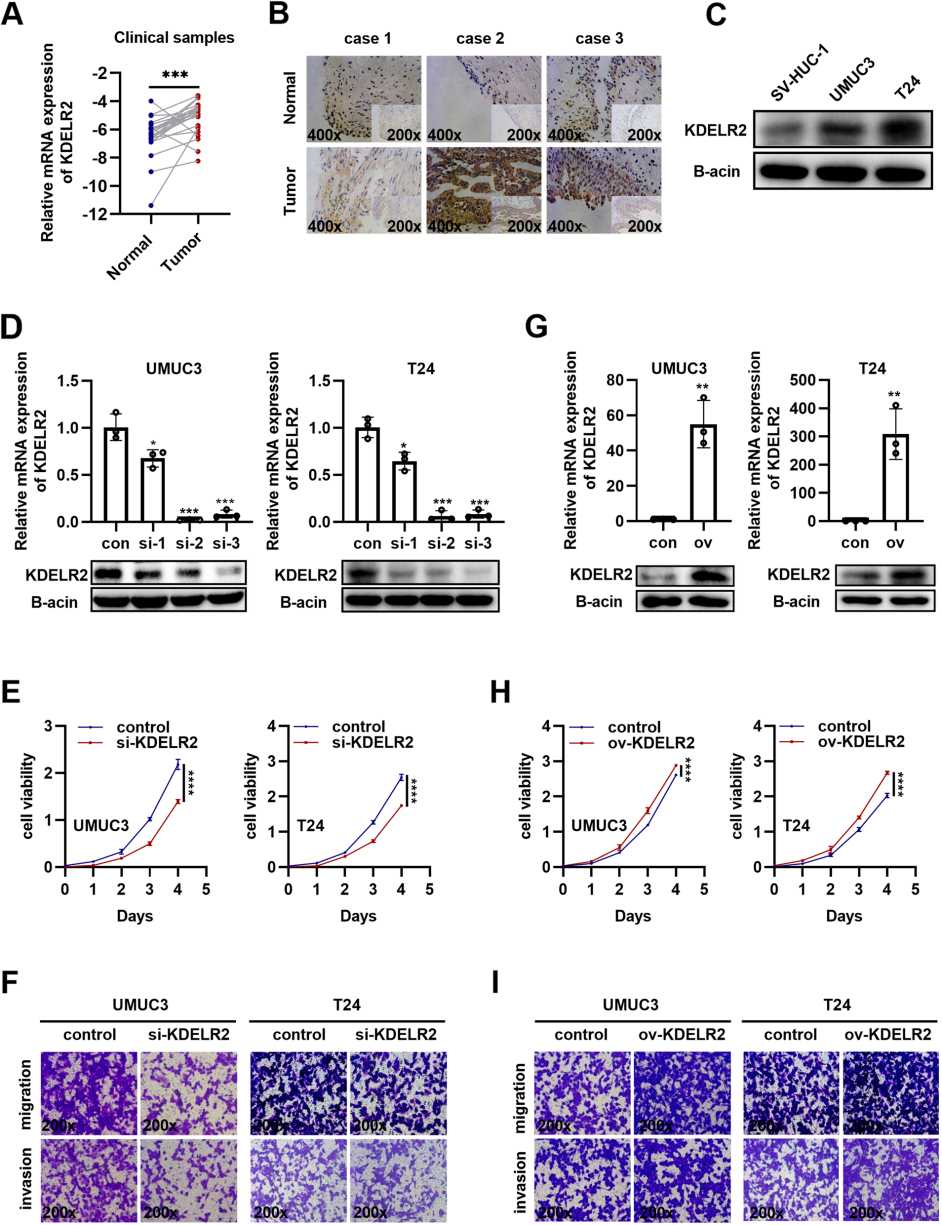
KDELR2 functions as an oncogene in BCa. **A-B** Verification of KDELR2 expression in clinical tissues by qRT-PCR (normal, *n* = 24; tumor, *n* = 24) and immunohistochemical (IHC) staining (200x, 400x). **C** Detection of KDELR2 expression by western blot analysis in cell lines (*n* = 3). **D**, **G** Identification of knockdown or overexpression efficiency of KDELR2 by qRT-PCR and western blot analysis (*n* = 3). **E**, **H** Proliferation analysis of UMUC3 or T24 cells in the control (con) and siKDELR2-3 or KDELR2-overexpressing (ov) group (*n* = 4). (F, I) Migration and invasion (200x) of BCa cells in the control and siKDELR2-3 or KDELR2-overexpressing group (*n* = 3). *p* < 0.05, *; *p* < 0.01, **; *p* < 0.001, ***; *p* < 0.0001, ****

In addition to the data obtained from publicly available clinical cohorts, we further verified the aberrant expression of KDELR2 in 24 clinical samples. The qRT-PCR assay indicated that KDELR2 was upregulated in BCa compared to normal bladder tissues, which was consistent with the results of the bioinformatic analysis (Fig. 2A). The ROC curves showed a similar high diagnostic efficiency of KDELR2 in the 24 clinical samples (sFig. 1E). Immunohistochemical analysis of the clinical samples revealed positive KDELR2 staining in the cytoplasm of tissues, indicating that KDELR2 is commonly overexpressed in BCa (Fig. 2B). In addition, higher expression levels of KDELR2 were detected in BCa cell lines (T24 and UMUC3) than in the urothelial cell line SV-HUC-1 (Fig. 2C, sFig. 1F), implying the potential role of KDELR2 in promoting bladder tumorigenesis. Subsequently, we evaluated the potential biological function of KDELR2 in BCa cells by introducing three independent siRNAs targeting KDELR2 into the UMUC3 and T24 cells. Interestingly, although all three siRNAs significantly inhibited the mRNA levels of KDELR2, only siKDELR2-3 significantly reduced the protein expression (Fig. 2D). Thus, siKDELR2-3 was introduced into BCa cells to observe cell growth. We found that KDELR2 suppression significantly reduced cell viability (Fig. 2E). Invasion and metastasis are hallmarks of cancer cells. KDELR malfunctions have been reported to be associated with changes in ECM degradation and cellular adhesion [14, 26]. We observed that the control cells had a high level of mobility and invasiveness than the siKDELR2 cells, indicating that siKDELR2 induced tumor suppression (Fig. 2F). We also established exogenous KDELR2-overexpressing T24 and UMUC3 cell lines to further understand the oncogenic functions of KDELR2 (Fig. 2G). Increased KDELR2 expression significantly increased the proliferation, migration, and invasion of BCa cells (Fig. 2H-I). Taken together, the results provide strong evidence that KDELR2 has oncogenic properties in vitro.

### KDELR2 is a positive regulator of MMP2, MMP9, MKI67 in BCa

**Fig. 3:**
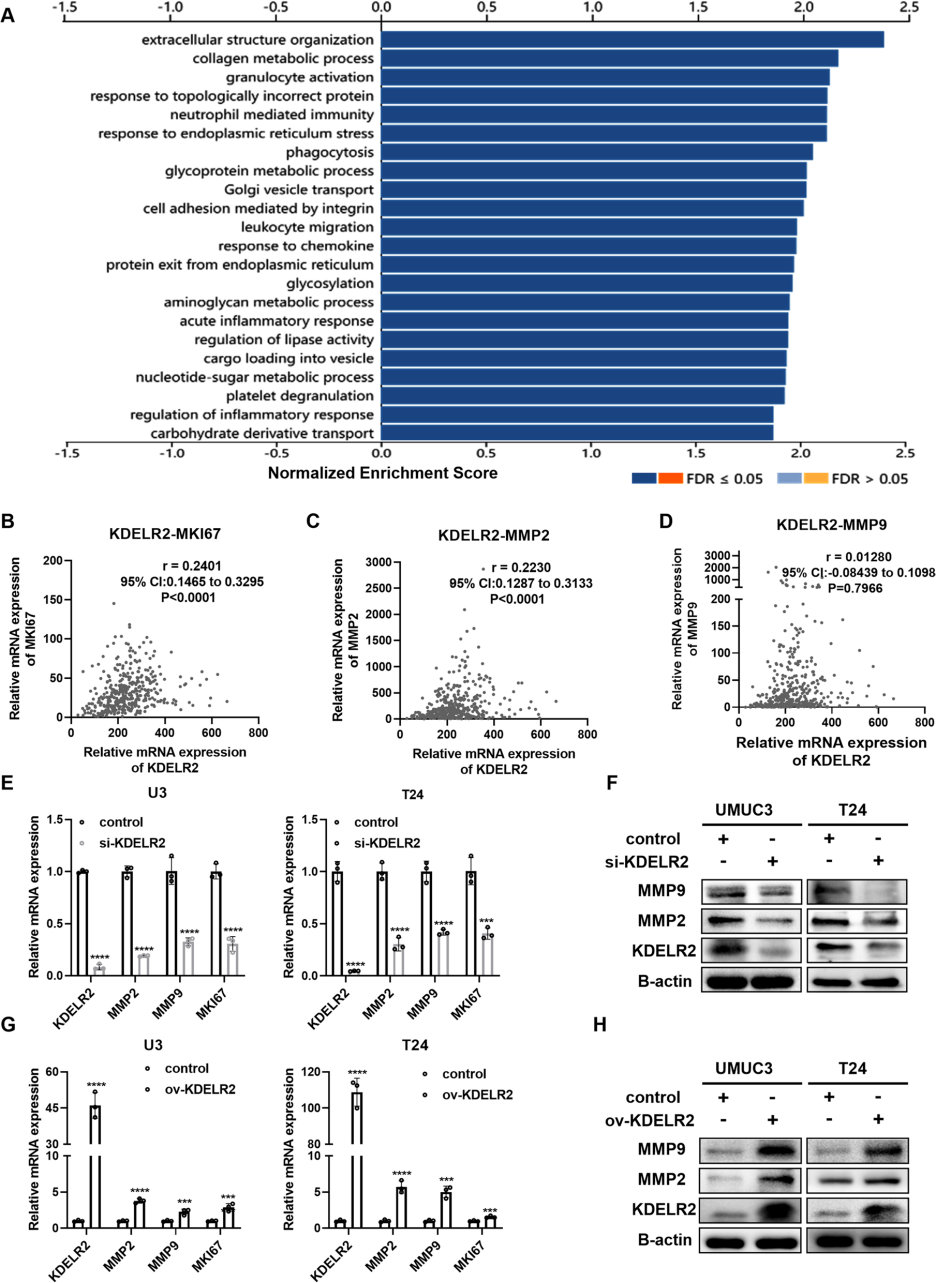
KDELR2 is a positive regulator of MMP2, MMP9, MKI67 in BCa. **A** Biological processes of KDELR2 mainly involved in BCa analysed using the GO and LinkedOmics tools. **B**-**D** The correlation of KDELR2 mRNA levels with MKI67, MMP2 and MMP9 in BCa (*n* = 408). **E**-**F** Detection of gene expression by qRT-PCR and western blot analysis in the siKDELR2 and control group UMUC3 and T24 cell lines (*n* = 3). **G**-**H** Detection of gene expression by qRT-PCR and western blot analysis in the KDELR2-overexpressing and control group UMUC3 and T24 cell lines (*n* = 3). *p* < 0.001, ***; *p* < 0.0001, ****

We performed GSEA to establish the potential biological functions of KDELR2 using the LinkedOmics tool. KEGG pathway enrichment analysis revealed that KDELR2 was related to protein processing in the ER, phagosome, ECM-receptor interaction, focal adhesion, cell adhesion molecules (CAMs), and regulation of the actin cytoskeleton (sFig. 2A). GO analysis showed that KDELR2 was mainly involved in the biological processes of immune responses, collagen metabolic process, phagocytosis and in regulating the ER stress and Golgi vesicle transport (Fig. 3A). To identify the components that assist KDELR2-mediated proliferation, we investigated its correlation with MKI67. We observed that the mRNA levels of KDELR2 positively correlated with those of MKI67 (r = 0.2401, p < 0.001) (Fig. 3B) in TCGA-BLCA dataset. In addition, we investigated some of the known proteases secreted by the Golgi, such as MMPs, to identify the components that assist KDELR2-mediated invasion. It has been well established that MMP2 [27] and MMP9 [28] enhance BCa invasion and metastasis. We observed a close correlation between KDELR2 and MMP2 (r = 0.2230, *p* < 0.001) (Fig. 3C). However, the correlation between KDELR2 and MMP9 was not statistically significant (r = 0.01280, *p* = 0.7966) (Fig. 3D). Next, we profiled the expression of these genes by RT-qPCR and western blot analysis after silencing or overexpressing KDELR2 in UMUC3 and T24 cells. The results revealed that altering KDELR2 regulated the expressions of MMP2, MMP9 and MKI67 in BCa cells (Fig. 3E-H). Taken together, our data suggest that KDELR2 regulates the expression of MMP2, MMP9 and MKI67 in BCa cells.

### Upregulation of KIF20A promotes tumor progression in BCa

**Fig. 4:**
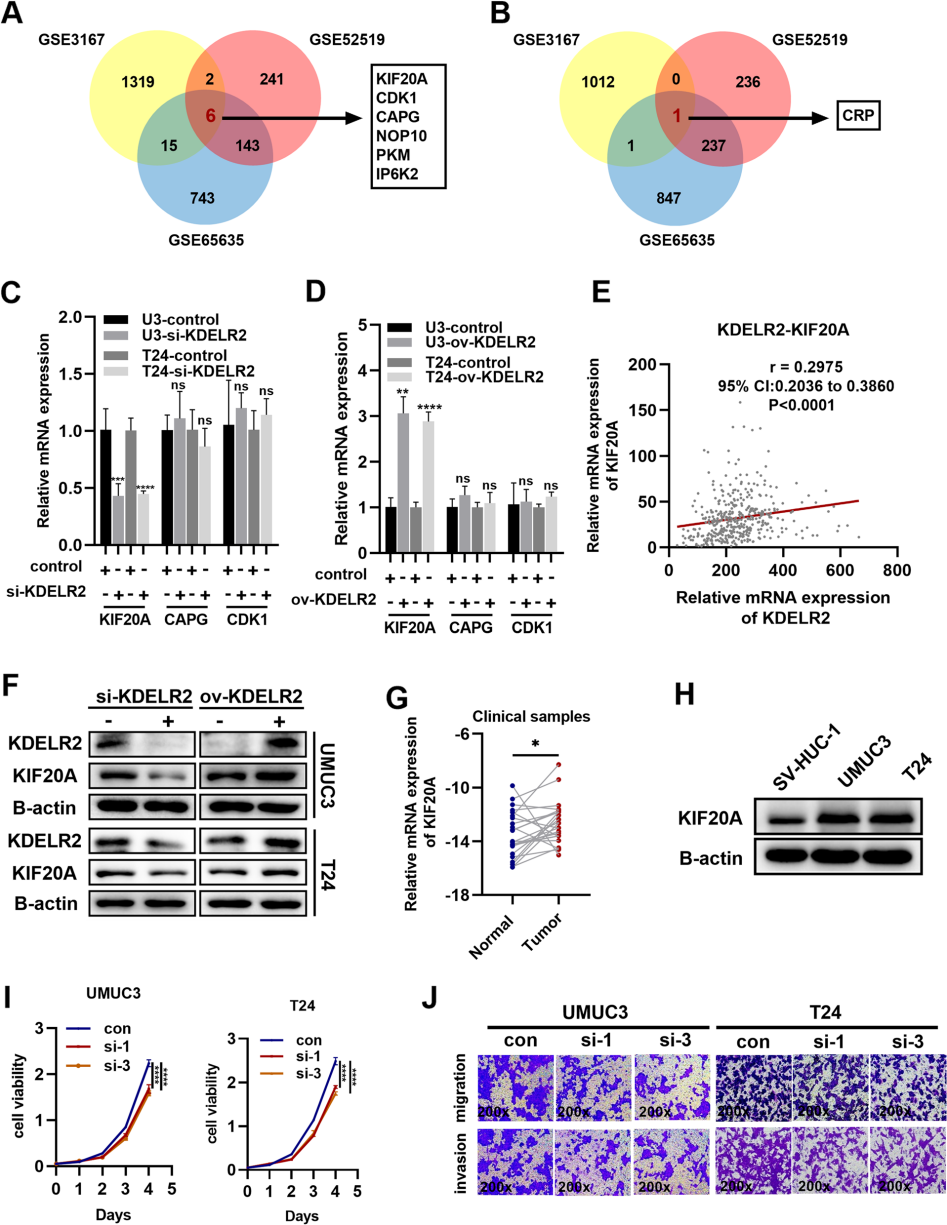
Upregulation of KIF20A promotes tumor progression in BCa. **A** A Venn diagram of the six commonly up-regulated DEGs (KIF20A, CDK1, CAPG, NOP10, PKM, IP6K2) in KDELR2 high expression group in the three independent GEO datasets (GSE3167, GSE52519 and GSE65635). **B** A Venn diagram of the commonly down-regulated DEGs (CRP) in KDELR2 low expression group. **C-D** Expression profiles of KIF20A, CAPG, and CDK1 mRNA in the siKDELR2 or KDELR2-overexpressing and control group UMUC3 and T24 cell lines (*n* = 3). **E** The correlation of KDELR2 mRNA levels with KIF20A in BCa (*n* = 408). **F** Detection of KDELR2 and KIF20A protein expressions by western blot analysis in the siKDELR2 or KDELR2-overexpressing group (*n* = 3). **G** Verification of KIF20A expression in clinical tissues by qRT-PCR (*n* = 24). **H** Detection of KIF20A expression by western blot analysis in cell lines (*n* = 3). **I** Proliferation analysis of UMUC3 or T24 cells in the control, siKIF20A-1, and siKIF20A-3 groups (*n* = 4). **J** Migration and invasion (200x) of BCa cells in the control, siKIF20A-1, and siKIF20A-3 groups (*n* = 3). *p* < 0.05, *; *p* < 0.01, **; *p* < 0.001, ***; *p* < 0.0001, ****

Furthermore, we observed that KDELR2 might be involved in gene regulation through regulation of epigenetic, gene silencing, RNA splicing or mRNA processing (sFig. 2B). Thus, we sought to investigate the regulatory effects of KDELR2 in BCa, and performed further bioinformatics analysis based on data from the GEO database. Based on the median value of KDELR2 expression, every GSE expression matrix was divided into high and low groups, and then limma package was used to accessed top DEGs that related to KDELR2. |Foldchange|> 1.5 and p-value < 0.05 were considered statistically significant. As shown in the Venn diagram, we identified six commonly up-regulated DEGs (KIF20A, CDK1, CAPG, NOP10, PKM, IP6K2) in KDELR2 high expression group (Fig. 4A) and one commonly down-regulated DEG (CRP) in KDELR2 low expression group (Fig. 4B). To further analyse the clinical significance of these prospective candidates in BCa, we evaluated their differential expression by analysing the TCGA-BLCA dataset. Accordingly, KIF20A, CAPG, and CDK1 were dysregulated in BCa, but no significant difference was observed in the other genes (sFig 2C). Importantly, we profiled the expression of KIF20A, CAPG, CDK1 by RT-qPCR analysis after silencing or overexpressing of KDELR2 in UMUC3 and T24 cells. The results revealed that altering KDELR2 regulated the expression of KIF20A, but not CAPG and CDK1 (Fig. 4C-D). We also observed that the mRNA levels of KIF20A were positively related to those of KDELR2 in BCa tissues (r = 0.2975, p < 0.001) (Fig. 4E). And changes in protein levels of KIF20A was also observed in BCa cells after altering KDELR2 (Fig. 4F). In addition, the ROC curves revealed that up-regulated KIF20 possess high diagnostic efficiency in BCa (sFig 2D). Thus, we identified KIF20A as a potential regulatory target of KDELR2 for further study.

To explore the underlying role of KIF20A in BCa, its abnormal expression in clinical samples and BCa cells were also evaluated. The results revealed that KIF20A was highly expressed in clinical samples and BCa cells (Fig. 4G-H), which implied that an abnormality in KIF20A might induce potential oncogene function in BCa. Similar to KDELR2, exogenous KIF20A and three independent siRNAs targeting KDELR2 were introduced into UMUC3 and T24 cells. The efficiency was profiled by RT-qPCR and western blot analysis (sFig. 3A-D). As expected, KIF20A knockdown markedly impaired the growth of UMUC3 and T24 cells (Fig. 4I), while its overexpression significantly increased cell proliferation (sFig. 3E). Transwell assays were performed to confirm the role of KIF20A as a driver of invasion and metastasis. KIF20A silencing significantly reduced cellular mobility and invasiveness (Fig. 4J), while higher KIF20A expression increased the invasiveness of UMUC3 and T24 cells compared with the control group (sFig. 3F). Collectively, these results suggest that upregulation of KIF20A promotes tumor progression in BCa.

### KDELR2 promotes BCa cell proliferation and metastasis in a KIF20A-mediated manner

**Fig. 5:**
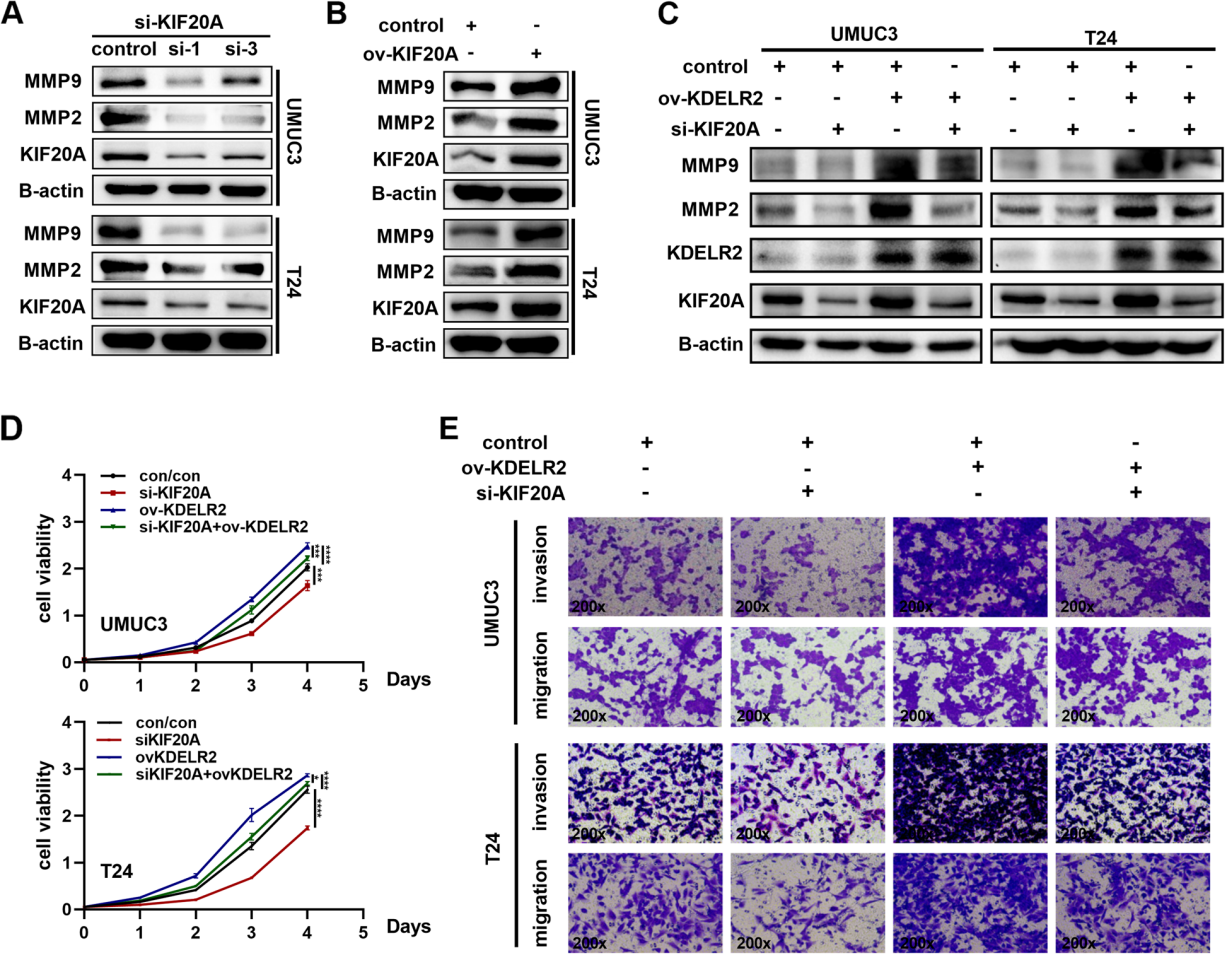
KDELR2 promotes BCa cell proliferation and metastasis in a KIF20A-mediated manner. **A**-**B** Detection of KIF20A, MMP2, MMP9 protein expressions by western blot analysis in the siKIF20A-1, siKIF20A-3 or KIF20A-overexpressing group (*n* = 3). **C** Knockdown of KIF20A could reverse the effect of KDELR2 overexpression on MMPs (*n* = 3). **D** CCK8 assays for the indicated cells (*n* = 4). **E** Migration and invasion assays (200x) for the indicated ccRCC cells (*n* = 3). *p* < 0.05, *; *p* < 0.01, **; *p* < 0.001, ***; *p* < 0.0001, ****

To investigate the underlying mechanism by which KIF20A drives BCa progression, we tested the expression levels of MMPs after altering KIF20A. As expected, overexpression and knockdown of KIF20A significantly upregulated and downregulated the protein expression of MMP2 and MMP9 in BCa cells, respectively (Fig. 5A-B). To further explore whether KDELR2 exerts regulatory effects via KIF20A, functional rescue experiments were performed. We evaluated the expression of MMP2 and MMP9 upon knockdown of KIF20A in KDELR2-amplified BCa cells. The results confirmed that silencing KIF20A in these cells reduced the levels of MMP2 and MMP9, thereby reducing its induction by KDELR2 (Fig. 5C). The CCK-8 assay results revealed that overexpression of KDERL2 promoted the proliferation of UMUC3 and T24 cells, which was nonetheless impaired by simultaneous knockdown of KIF20A (Fig. 5D). Transwell assays also revealed that KIF20A knockdown partially attenuated the effects of overexpression of KDELR2 on BCa cell metastasis compared with that of the controls (Fig. 5E), indicating that KDELR2 promotes BCa cell proliferation and metastasis in a KIF20A-mediated manner in vitro.

### KDELR2 is important for BCa progression in vivo

**Fig. 6:**
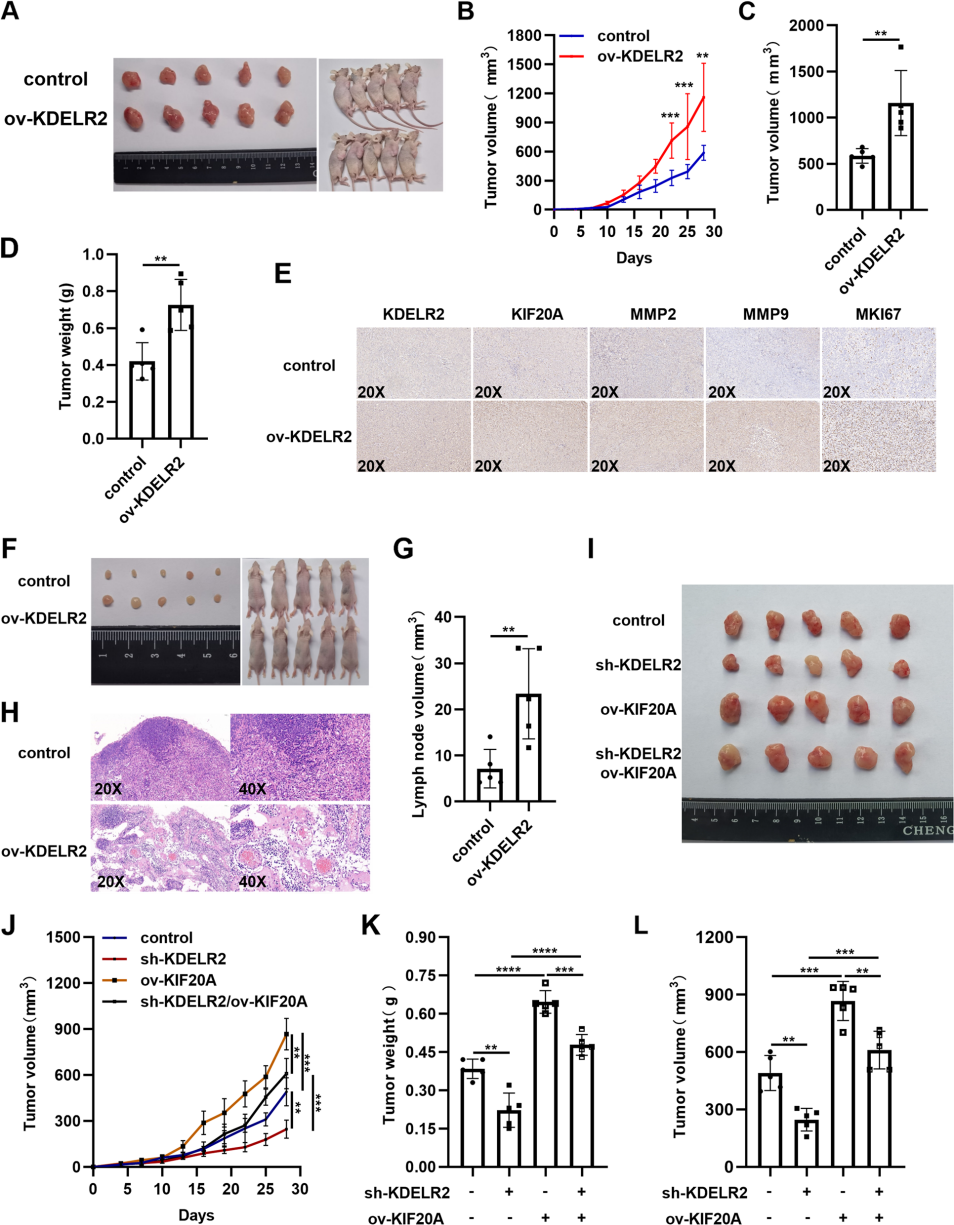
Upregulation of KDELR2 and KIF20A promotes tumor progression in vivo. **A-D** Subcutaneous tumor in mice, its growth curve and final volume and weight (*n* = 5). **E** IHC staining (20x) for KDELR2, KIF20A, MMP2, MMP9 and MKI67. **F-G** Footpad tumor in mice as well as popliteal LNs and volume (*n* = 5). **H** HE staining (20x, 40x) of the popliteal LNs. **I-L** Subcutaneous tumor in mice, its growth curve and final volume and weight (*n* = 5). *p* < 0.05, *; *p* < 0.01, **; *p* < 0.001, ***

To further confirm the aforementioned in vitro findings, T24 cells stably transfected with KDELR2 or control vector were subcutaneously injected into BALB/c nude mice. Supporting the results obtained in vitro, the xenograft experiments showed that the tumor volume and weight significantly increased in the KDELR2 overexpression group (Fig. 6A-D). As evidenced by IHC staining, the expression levels of KIF20A, MMP-2, MMP-9 and MKI67 increased significantly in tumor tissues in the KDELR2 overexpression group (Fig. 6E). Furthermore, T24 cells were injected into the footpads of nude mice to construct a lymph node metastasis model to confirm the role of KDELR2 in facilitating tumor metastasis (Fig. 6F). We found that the volumes of popliteal LNs increased in the stable KDELR2 overexpression group (Fig. 6G). HE staining of the popliteal LNs further indicated that upregulation of KDELR2 could promote lymphatic metastasis of BCa in vivo (Fig. 6H). We also observed a significant reduction in tumor volume and weight in the stable KDELR2 silence group (Fig. 6I-L). Additionally, overexpression of KIF20A resulted in an increased tumor growth, which also reversed KDELR2 silence-induced decreased tumor growth (Fig. 6I-L). Taken together, the present study indicated that KDELR2 facilitated the expression of KIF20A, MMP-2, MMP-9 and MKI67 and BCa growth and metastasis in vivo.

## Discussion

Postoperative recurrence and distant metastasis remain a conundrum for BCa prognosis. Therefore, understanding the regulators of these processes is important to therapeutically prevent BCa metastasis. KDELR2 has been reported to be a robust and independent driver of lung cancer invasion and metastasis [15]. In the current study, we revealed that KDELR2 was highly expressed in BCa, especially advanced BCa tissues. In addition, we identified KDELR2 as a potential risk factor with prognostic value in patients with BCa, especially those harbouring the KDRLR2 amplification. Moreover, bioinformatic analysis with a gain-of-function invasion screen revealed that KDELR2 was a robust and independent driver of BCa cell proliferation and tumorigenicity. Our data indicated that KDELR2 could alter cellular proliferation and robustly promote invasion and metastasis.

There has been a long-established connection between cancer outcomes and the transport homeostasis between the Golgi and the ER; however, mechanistic studies remain limited. After KDELR/ligand interaction, the active G_α_ subunits activate their specific target protein kinases, which subsequently modulate gene transcription followed by regulation of the anterograde or retrograde trafficking [10, 29]. Our results provide the first evidence that active KDELR2 regulates the transcription and expression of MMP2, MMP9 and MKI67. Previous reports showed that MMP secretion was modulated by alterations in KDELR2. RT-qPCR revealed that the MMPs 1a, 2 and 9 were transcriptionally upregulated upon KDELR2 overexpression; however, they did not show any change upon gene repression [15]. Interestingly, in this study, we observed alterations in mRNA and protein expression in both MMP2 and MMP9 upon KDELR2 overexpression or repression. Thus, the tumor-promotive function of KDELR2 on MMP2 and MMP9 might be tissue/cell-specific. Detailed parallel studies on different tissue/cell types are required to further elucidate the complex functionality.

Despite evidence highlighting the roles of KIF20A in various tumor cells and its importance in regulating tumorigenesis, the underlying mechanism in BCa remains largely elusive. KIF20A could promote the growth of colorectal cancer cells via the JAK/STAT3 signalling pathway [30]. Moreover, inhibiting KIF20A resulted in gastric cancer cell mitosis (G2/M phase) arrest and improved drug resistance to chemotherapy [20]. However, KIF20A overexpression conferred resistance to paclitaxel in breast cancer cases [9]. Collectively, the results demonstrated that KIF20A played a critical role in the proliferation, invasion and chemotherapeutic drug sensitivity of the tumor cells. Although abnormal expression of KIF20A has been reported in BCa, the present study is the first to reveal its specific mechanism in promoting tumor progression and regulating the expression of MMPs. Mechanistically, this study identified KIF20A as a regulatory target of KDELR2, in which KDELR2 promotes KIF20A expression and KIF20A enhances MMP2 and MMP9 expression to drive cellular invasion and metastasis. Therefore, our findings elucidate a hitherto unexplored mechanism of KDELR2 and KIF20A in BCa, linking a Golgi‐ER traffic transport protein to a critical kinesin during cancer progression.

MMP2 and MMP9 are two major MMPs that play critical roles in the degradation of various ECM components. Both two MMPs are overexpressed in many cancers, which correlate with increased cancer cell invasion and metastases [31, 32]. Interestingly, MMP2 and MMP9 have been studied extensively considering their prognostic values and pro‐invasive functions in BCa [28, 33]. The results of the current study showed that the expression levels of MMP2 and MMP9 correlated well with KIF20A and KDELR2 overexpression and BCa cell invasion. The experiments further revealed that both KIF20A and KDELR2 upregulated the transcription and expression of MMP2 and MMP9, further promoting the invasion of BCa cells.

Nevertheless, our research has certain limitations. We conducted the survival analysis on KDELR2 using a reliable public database; however, additional studies are needed to evaluate the role of KDELR2 in patients with BCa. In subsequent work, we will enlarge the KDELR2 sample amount and supplement sufficient follow-up information of clinical patients. Furthermore, additional studies are needed for an in-depth investigation of the mechanism by which KDELR2 promotes the expression of KIF20A.

## Conclusions

In summary, our study elucidated the individual roles of KDELR2 and KIF20A in driving BCa tumorigenesis and metastasis. In addition, our study revealed that the combined inhibition of KDELR2 and KIF20A could be a promising therapeutic option in patients with BCa. Our findings may have broader implications for targeted therapies that could be extended to other cancers.

## Materials and methods

### Human BCa and paired adjacent specimens

Human BCa tumor samples were obtained from the Department of Urology, Union Hospital, Tongji Medical College, Wuhan, China. The study complied with the relevant ethical regulations regarding research involving human participants. Written informed consent was obtained from each participant, and the local ethics committee approved this study.

### Cell culture and reagents

The T24, UMUC3 and SV-HUC-1 cells were obtained from the American Type Culture Collection (ATCC, Manassas, VA). Cells were cultured in DMEM (for UMUC3), 1640 (for T24) and F12K (for SV-HUC-1) mediums supplemented with fetal bovine serum (10%) and incubated in 5% CO2 at 37 °C.

### Gene expression and gene silencing

UMUC3 and T24 cells were cultured in 6-well plates with approximately 60%–70% confluence. KDELR2 and KIF20A‐specific overexpression plasmids were obtained from Genechem, China. Cells were transfected with 3 µg of the overexpression plasmids or controlled plasmid using 3 ul Lipofectamine 3000 (Invitrogen, CA, USA) per well. The lentivirus-transduced stably expressed KDELR2 or control was structured by Generulor Company Bio-X Lab (Zhuhai, Guangdong, China). Briefly, KDELR2‐specific overexpression plasmids and helper plasmids were transiently co‐transfected into highly transfectable 293 T cells to generate high-titre lentivirus. The lentivirus-transduced stably expressed KIF20A or control, and the shRNA for KDELR2 (target sequences: GTCCAGACCATCCTATACT) or control was structured by Genechem, China. The lentiviral transduction system contained 25 ul of the transfection reagent A/P (Genechem, China) and 40 MOI lentivirus.

KDELR2 and KIF20A siRNAs were obtained from GenePharma, China. Cells were transfected with 5 ul KDELR2/KIF20A siRNA (10 µM) or siRNA control using 3 ul Lipofectamine RNAiMAX (Invitrogen, Thermo Fisher Scientific Inc.) per well. The sequences of the siRNA fragments used are as follows: siKDELR2-1 (KDELR2-homo-395): 5′-CCGCAGCUAUUUAUGAUCATT-3′, 5′-UGAUCAUAAAUAGCUGCGGTT-3′; siKDELR2-2 (KDELR2-homo-466): 5′-CCUCUAUCGUGCUUUGUAUTT-3′, 5′- AUACAAAGCACGAUAGAGGTT-3′; siKDELR2-3 (KDELR2-homo-554): 5′-GUCCAGACCAUCCUAUACUTT-3′, 5-AGUAUAGGAUGGUCUGGACTT-3′; siKIF20A-1 (KIF20A-homo-1958): 5′-GCAUCUACCUAUGAUGAAATT-3′, 5′-UUUCAUCAUAGGUAGAUGCTT-3′; siKIF20A-2 (KIF20A-homo-2823), 5′-CCACUUGUGAUGACAUCUUTT-3′, 5′-AAGAUGUCAUCACAAGUGGTT-3′; siKIF20A-3 (KIF20A-homo-3068), 5′-CCAACCUGCCAAAGCUCAATT-3′, 5′-UUGAGCUUUGGCAGGUUGGTT-3′. Subsequently, the cells were collected after incubation for 48 h. The efficiency of silencing and overexpression was validated by qPCR and western blot analysis.

### Cell proliferation assays

The cell proliferation assay was performed based on a method described previously [34]. Briefly, 2000 cells were plated in 96-well plates, and the cell proliferation rates were measured using the MTS method according to the manufacturer's instructions.

### Transwell assays

The migration assays were performed based on a method described previously [35]. Briefly, after pretreatment with serum-free medium for 24 h, 50,000 UMUC3 and T24 cells were seeded in the top chamber (8 μm pore size, Corning). After 12 h (T24) and 24 h (UMUC3), the cells on the lower surface were fixed in 100% methanol and stained with 0.05% crystal violet, photographed randomly (200x) and counted. A similar protocol was followed for the invasion assays, except that the cells were further required to invade through Matrigel (Thermo Fisher Scientific).

### RNA isolation and qRT-PCR

Total RNA was extracted using the TRIzol reagent (Thermo, Massachusetts, USA) and reverse-transcribed as described previously [36]. The real-time PCR analysis was performed using SYBR Green mix (Thermo, Massachusetts, USA). Results were normalised to GAPDH in each sample. Gene primers were obtained from Sangon Biotech (Shanghai) and are listed:

- GAPDH
- Forward 5′‐GAGTCAACGGATTTGGTCGT‐3′
- Reverse 5′‐GACAAGCTTCCCGTTCTCAG‐3.
- KDELR2
- Forward5′‐ACCTTCCGAGTGGAGTTTCTGG‐3′
- Reverse5′‐ATAGCCACGGACTCCAGGTAGA‐3.
- KIF20A
- Forward5′‐GCCGCAGTCACAGCATCTTCTC‐3′
- Reverse5′‐TTCCTTCAACCGTTCACCACTCTTC‐3.
- MKI67
- Forward5′‐CCAAGCCACAGTCCAAGAGAAGTC‐3′
- Reverse5′‐TGCTGATGGTGTTCGTTTCCTGAG‐3.
- MMP2
- Forward5′‐CACCTACACCAAGAACTTCCGTCTG‐3′
- Reverse5′‐GTGCCAAGGTCAATGTCAGGAGAG‐3.
- MMP9
- Forward5′‐CGGTTTGGAAACGCAGATGG‐3′
- Reverse5′‐CGGAGTAGGATTGGCCTTGG‐3.
- CAPG
- Forward5′‐GCAGCTCTGTATAAGGTCTCTGA‐3′
- Reverse5′‐TTTCGCCCCTTCCAGATATAGA‐3.
- CDK1
- Forward5′‐AAACTACAGGTCAAGTGGTAGCC‐3′
- Reverse5′‐TCCTGCATAAGCACATCCTGA‐3.

### Western blot

The assays were performed as described previously [37]. Antibody recognizing KDELR2 (1:1000, DF4047) was obtained from Affinity Biosciences, China. Antibodies for KIF20A (1:1000, A15377) and β-Actin (1:10,000, AC004) were obtained from ABclonal. Antibodies for MMP2 (1:1000, GB11130) and MMP9 (1:1000, GB11132) were obtained from Servicebio, Wuhan, China.

### Animal experiments

BALB/c nude mice (5–6 weeks old) were purchased from Beijing HFK Bioscience (Beijing, China). All animal care and experimental procedures were approved by the Institutional Animal Use and Care Committee of Tongji Medical College, Huazhong University of Science and Technology and were performed according to established guidelines. The study complied with the relevant ethical regulations pertaining to animal research.

For the tumor growth study, 5 × 10^6^ T24 cells were injected subcutaneously into the flank of mice. Each group contained five mice. Tumor size was measured every three days. Four weeks later, the mice were sacrificed, and the weight and volume of the tumors were assessed.

For the popliteal lymph node (LN) metastasis assay, 10 mice were randomly assigned to two group. 5 × 10^5^ lentivirus-transduced stably expressed KDELR2 or control T24 cells were inoculated into the footpads. After 5 weeks, the popliteal LNs were enucleated and measured. The tumor and LN volumes were measured using the following formula: length × width^2 ^× 0.52. Tumor specimens and popliteal LNs were fixed and embedded in paraffin for HE staining and immunohistochemical (IHC) analysis.

### Analysis of differentially expressed genes

The gene expression profiles in three BCa datasets, including GSE3167 [38], GSE52519 [39], and GSE65635 [39], were obtained from the Gene Expression Omnibus (GEO) database. GSE3167 contained 41 BCa and 19 normal tissue samples. GSE52519 contained 3 BCa and 9 normal tissue samples. GSE65635 contained 4 BCa and 8 normal tissue samples. The differentially expressed genes (DEGs) between BCa and normal samples were screened using GEO2R. DEGs with log_2_FoldChange (logFC) ≥ 1.0 (upregulated) or logFC ≤  − 1.0 (downregulated) and p-value < 0.05 were considered statistically significant.

### Bioinformatic analysis

The UCSC Xena browser (https://xenabrowser.net/) was used to investigate the gene expression profiles and the correlation of between KDELR2 and KIF20A, MMP2, MMP9, and MKI67 in bladder urothelial carcinoma (BLCA) from The Cancer Genome Atlas (TCGA) database. The normalisation of gene expression was performed using the edgeR package. The gene expression profiles of KIF20A, CDK1, CAPG, NOP10, PKM, IP6K2, and CRP was obtained from the GEPIA [40]. TCGA-BLCA dataset contained 414 BCa tissue samples and 19 non-cancerous samples. The clinical information and pathology records of the patients with BCa were also obtained from TCGA-BLCA database and were used to evaluate the overall survival (OS) and disease-free survival (DFS) of the patients. The GSE32894 dataset [41], contained the clinical information and gene expression profile of 224 patients with BCa, which was also used for the survival analysis.

To identify the underlying biological functions of KDELR2, Gene Set Enrichment Analysis (GSEA) was performed in the LinkInterpreter module of the LinkedOmics tool [42]. GO assessed the biological processes (BP). The minimum number of genes (size) was set at 10 and simulations as 500. The P-value cut-off was set at 0.05.

### Statistical analysis

GraphPad Prism 8.0 (GraphPad Software Inc.) was used to perform the statistical analyses. Results are presented as means ± standard error of the mean (SEM). Student’s t-test or one-way analysis of variance (ANOVA) was used to evaluate the differences in KDELR2 gene expression between different subgroups of patients. In addition, the Pearson correlation coefficient and chi-squared test were performed to assess the associations between KDELR2 expression levels and clinicopathological characteristics. Receiver operating characteristic (ROC) curves were plotted, and the area under the curve (AUC) was calculated to analyse the diagnostic efficiency. The prognostic significance of KDELR2 to predict the OS and DFS of patients with BCa were estimated by the Kaplan–Meier method. Furthermore, the log-rank test was used to depict the OS or PFS distributions of the patients with different expression levels of KDELR2. A confidence threshold, *p* < 0.05, was considered statistically significant. *, *p* < 0.05; **, *p* < 0.01; ***, *p* < 0.001; ****, *p* < 0.0001.

### Data Availability

The data analyzed in this study were obtained from Gene Expression Omnibus (GEO) at GSE3167, GSE52519, GSE65635. The gene expression profiles were obtained from The Cancer Genome Atlas (TCGA) database. The datasets used and/or analyzed during the current study are available from the corresponding author on reasonable request.

## Supplementary Information

**Additional file 1: Figure S1.** KDELR2 has a prognostic and diagnostic value in BCa. **A**-**B** Receiver operating characteristic (ROC) curves of KDELR2 in the GES3167 (normal, *n* = 19; tumor, *n* = 41) and TCGA-BLCA datasets (normal, *n* = 19; tumor, n = 408). (C) Kaplan–Meier curves of KDELR2 expression in patients with BCa in the GSE32894 dataset (high, *n* = 112; low, *n* = 112). (D) Multivariate analysis of KDELR2 mRNA level and DFS in patients with BCa. **E** ROC curves of KDELR2 in clinical samples (normal, *n* = 24; tumor, *n* = 24). **F** Detection of KDELR2 expression by qRT-PCR analysis in cell lines (*n* = 4). *p* < 0.001, ***; *p* <0.0001, ****.

**Additional file 2: Figure S2.** **A**-**B** Biological processes of KDELR2 mainly involved in BCa analysed by KEGG pathway enrichment and GO analysis with the LinkedOmics tool. **C** Expression profiles of KIF20A, CDK1, CAPG, NOP10, PKM, IP6K2, and CRP mRNA in the TCGA-BLCA datasets (normal, *n* = 19; tumor, *n* = 408). **D** ROC curves of KIF20A in TCGA-BLCA datasets (normal, *n* = 19; tumor, n = 408). *p* < 0.05, *.

**Additional file 3: Figure S3.** Identify the expression of KIF20A and its biological function. **A**-**D** Identification of knockdown or overexpression efficiency of KIF20A by qRT-PCR and western blot analysis (*n* = 3). **E** Proliferation analysis of UMUC3 or T24 cells in the control and KIF20A-overexpressing groups (*n* = 4). **F** Migration and invasion (200x) of BCa cells in the control and KIF20A-overexpressing groups (*n* = 3). *p* < 0.01, **; *p* < 0.001, ***; *p* <0.0001, ****.

## Data Availability

The study was based on the data available at TCGA (https://www.cancer.gov/tcga) and the Gene Expression Omnibus (GEO) database (http://www.ncbi.nlm.nih.gov/geo). The datasets used and/or analyzed during the current study are available from the corresponding author on reasonable request.

## Abbreviations

BCa: Bladder cancer
KDELR2: KDEL endoplasmic reticulum protein retention receptor 2
KIF20A: Kinesin family member 20A
MMPs: Matrix metalloproteinases
MKI67: Marker of proliferation Ki-67
ER: Endoplasmic reticulum
BLCA: Bladder urothelial carcinoma
TCGA: The Cancer Genome Atlas database
OS: Overall survival
DFS: Disease-free survival
GSEA: Gene Set Enrichment Analysis
ROC: Receiver operating characteristic

## References

[1] Kamat AM, Hahn NM, Efstathiou JA, Lerner SP, Malmstrom PU, Choi W, Guo CC, Lotan Y, Kassouf W (2016). Bladder cancer. Lancet.

[2] Sung H, Ferlay J, Siegel RL, Laversanne M, Soerjomataram I, Jemal A, Bray F (2021). Global Cancer Statistics 2020: GLOBOCAN Estimates of Incidence and Mortality Worldwide for 36 Cancers in 185 Countries. CA Cancer J Clin.

[3] Siegel RL, Miller KD, Fuchs HE, Jemal A (2021). Cancer Statistics, 2021. CA Cancer J Clin.

[4] Nadal R, Bellmunt J (2019). Management of metastatic bladder cancer. Cancer Treat Rev.

[5] Capitani M, Sallese M (2009). The KDEL receptor: new functions for an old protein. FEBS Lett.

[6] Raykhel I, Alanen H, Salo K, Jurvansuu J, Nguyen VD, Latva-Ranta M, Ruddock L (2007). A molecular specificity code for the three mammalian KDEL receptors. J Cell Biol.

[7] Ruggiero C, Fragassi G, Grossi M, Picciani B, Di Martino R, Capitani M, Buccione R, Luini A, Sallese M (2015). A Golgi-based KDELR-dependent signalling pathway controls extracellular matrix degradation. Oncotarget.

[8] Xiu G, Sui X, Wang Y, Zhang Z (2018). FOXM1 regulates radiosensitivity of lung cancer cell partly by upregulating KIF20A. Eur J Pharmacol.

[9] Khongkow P, Gomes AR, Gong C, Man EP, Tsang JW, Zhao F, Monteiro LJ, Coombes RC, Medema RH, Khoo US (2016). Paclitaxel targets FOXM1 to regulate KIF20A in mitotic catastrophe and breast cancer paclitaxel resistance. Oncogene.

[10] Giannotta M, Ruggiero C, Grossi M, Cancino J, Capitani M, Pulvirenti T, Consoli GM, Geraci C, Fanelli F, Luini A (2012). The KDEL receptor couples to Galphaq/11 to activate Src kinases and regulate transport through the Golgi. EMBO J.

[11] Kamimura D, Katsunuma K, Arima Y, Atsumi T, Jiang JJ, Bando H, Meng J, Sabharwal L, Stofkova A, Nishikawa N (2015). KDEL receptor 1 regulates T-cell homeostasis via PP1 that is a key phosphatase for ISR. Nat Commun.

[12] Liao Z, She C, Ma L, Sun Z, Li P, Zhang X, Wang P, Li W (2019). KDELR2 Promotes Glioblastoma Tumorigenesis Targeted by HIF1a via mTOR Signaling Pathway. Cell Mol Neurobiol.

[13] Mao H, Nian J, Wang Z, Li X, Huang C (2020). KDELR2 is an unfavorable prognostic biomarker and regulates CCND1 to promote tumor progression in glioma. Pathol Res Pract.

[14] Ruggiero C, Grossi M, Fragassi G, Di Campli A, Di Ilio C, Luini A, Sallese M (2018). The KDEL receptor signalling cascade targets focal adhesion kinase on focal adhesions and invadopodia. Oncotarget.

[15] Bajaj R, Kundu ST, Grzeskowiak CL, Fradette JJ, Scott KL, Creighton CJ, Gibbons DL (2020). IMPAD1 and KDELR2 drive invasion and metastasis by enhancing Golgi-mediated secretion. Oncogene.

[16] Taniuchi K, Nakagawa H, Nakamura T, Eguchi H, Ohigashi H, Ishikawa O, Katagiri T, Nakamura Y (2005). Down-regulation of RAB6KIFL/KIF20A, a kinesin involved with membrane trafficking of discs large homologue 5, can attenuate growth of pancreatic cancer cell. Cancer Res.

[17] Kikuchi T, Daigo Y, Katagiri T, Tsunoda T, Okada K, Kakiuchi S, Zembutsu H, Furukawa Y, Kawamura M, Kobayashi K (2003). Expression profiles of non-small cell lung cancers on cDNA microarrays: identification of genes for prediction of lymph-node metastasis and sensitivity to anti-cancer drugs. Oncogene.

[18] Zhao X, Zhou LL, Li X, Ni J, Chen P, Ma R, Wu J, Feng J (2018). Overexpression of KIF20A confers malignant phenotype of lung adenocarcinoma by promoting cell proliferation and inhibiting apoptosis. Cancer Med.

[19] Groth-Pedersen L, Aits S, Corcelle-Termeau E, Petersen NH, Nylandsted J, Jaattela M (2012). Identification of cytoskeleton-associated proteins essential for lysosomal stability and survival of human cancer cells. PLoS ONE.

[20] Yan GR, Zou FY, Dang BL, Zhang Y, Yu G, Liu X, He QY (2012). Genistein-induced mitotic arrest of gastric cancer cells by downregulating KIF20A, a proteomics study. Proteomics.

[21] Gasnereau I, Boissan M, Margall-Ducos G, Couchy G, Wendum D, Bourgain-Guglielmetti F, Desdouets C, Lacombe ML, Zucman-Rossi J, Sobczak-Thepot J (2012). KIF20A mRNA and its product MKlp2 are increased during hepatocyte proliferation and hepatocarcinogenesis. Am J Pathol.

[22] Shen T, Yang L, Zhang Z, Yu J, Dai L, Gao M, Shang Z, Niu Y (2019). KIF20A Affects the Prognosis of Bladder Cancer by Promoting the Proliferation and Metastasis of Bladder Cancer Cells. Dis Markers.

[23] Stangel D, Erkan M, Buchholz M, Gress T, Michalski C, Raulefs S, Friess H, Kleeff J (2015). Kif20a inhibition reduces migration and invasion of pancreatic cancer cells. J Surg Res.

[24] Taniuchi K, Furihata M, Saibara T (2014). KIF20A-mediated RNA granule transport system promotes the invasiveness of pancreatic cancer cells. Neoplasia.

[25] Sheng Y, Wang W, Hong B, Jiang X, Sun R, Yan Q, Zhang S, Lu M, Wang S, Zhang Z (2018). Upregulation of KIF20A correlates with poor prognosis in gastric cancer. Cancer Manag Res.

[26] Blum A, Khalifa S, Nordstrom K, Simon M, Schulz MH, Schmitt MJ (2019). Transcriptomics of a KDELR1 knockout cell line reveals modulated cell adhesion properties. Sci Rep.

[27] Kader AK, Liu J, Shao L, Dinney CP, Lin J, Wang Y, Gu J, Grossman HB, Wu X (2007). Matrix metalloproteinase polymorphisms are associated with bladder cancer invasiveness. Clin Cancer Res.

[28] Liu F, Zhang H, Xie F, Tao D, Xiao X, Huang C, Wang M, Gu C, Zhang X, Jiang G (2020). Hsa_circ_0001361 promotes bladder cancer invasion and metastasis through miR-491-5p/MMP9 axis. Oncogene.

[29] Pulvirenti T, Giannotta M, Capestrano M, Capitani M, Pisanu A, Polishchuk RS, San Pietro E, Beznoussenko GV, Mironov AA, Turacchio G (2008). A traffic-activated Golgi-based signalling circuit coordinates the secretory pathway. Nat Cell Biol.

[30] Xiong M, Zhuang K, Luo Y, Lai Q, Luo X, Fang Y, Zhang Y, Li A, Liu S (2019). KIF20A promotes cellular malignant behavior and enhances resistance to chemotherapy in colorectal cancer through regulation of the JAK/STAT3 signaling pathway. Aging (Albany NY).

[31] Yan L, Lin B, Gao L, Gao S, Liu C, Wang C, Wang Y, Zhang S, Iwamori M (2010). Lewis (y) antigen overexpression increases the expression of MMP-2 and MMP-9 and invasion of human ovarian cancer cells. Int J Mol Sci.

[32] Chen JS, Huang XH, Wang Q, Huang JQ, Zhang LJ, Chen XL, Lei J, Cheng ZX (2013). Sonic hedgehog signaling pathway induces cell migration and invasion through focal adhesion kinase/AKT signaling-mediated activation of matrix metalloproteinase (MMP)-2 and MMP-9 in liver cancer. Carcinogenesis.

[33] Yang R, Xu J, Hua X, Tian Z, Xie Q, Li J, Jiang G, Cohen M, Sun H, Huang C (2020). Overexpressed miR-200a promotes bladder cancer invasion through direct regulating Dicer/miR-16/JNK2/MMP-2 axis. Oncogene.

[34] Xiao W, Xiong Z, Xiong W, Yuan C, Xiao H, Ruan H, Song Z, Wang C, Bao L, Cao Q (2019). Melatonin/PGC1A/UCP1 promotes tumor slimming and represses tumor progression by initiating autophagy and lipid browning. J Pineal Res.

[35] Meng X, Xiong Z, Xiao W, Yuan C, Wang C, Huang Y, Tong J, Shi J, Chen Z, Liu C (2020). Downregulation of ubiquitin-specific protease 2 possesses prognostic and diagnostic value and promotes the clear cell renal cell carcinoma progression. Ann Transl Med.

[36] Xiao W, Wang C, Chen K, Wang T, Xing J, Zhang X, Wang X (2020). MiR-765 functions as a tumour suppressor and eliminates lipids in clear cell renal cell carcinoma by downregulating PLP2. EBioMedicine.

[37] Xiong Z, Xiong W, Xiao W, Yuan C, Shi J, Huang Y, Wang C, Meng X, Chen Z, Yang H (2021). NNT-induced tumor cell "slimming" reverses the pro-carcinogenesis effect of HIF2a in tumors. Clin Transl Med.

[38] Dyrskjot L, Kruhoffer M, Thykjaer T, Marcussen N, Jensen JL, Moller K, Orntoft TF (2004). Gene expression in the urinary bladder: a common carcinoma in situ gene expression signature exists disregarding histopathological classification. Cancer Res.

[39] Borisov N, Tkachev V, Suntsova M, Kovalchuk O, Zhavoronkov A, Muchnik I, Buzdin A (2018). A method of gene expression data transfer from cell lines to cancer patients for machine-learning prediction of drug efficiency. Cell Cycle.

[40] Tang Z, Li C, Kang B, Gao G, Li C, Zhang Z (2017). GEPIA: a web server for cancer and normal gene expression profiling and interactive analyses. Nucleic Acids Res.

[41] Sjodahl G, Lauss M, Lovgren K, Chebil G, Gudjonsson S, Veerla S, Patschan O, Aine M, Ferno M, Ringner M (2012). A molecular taxonomy for urothelial carcinoma. Clin Cancer Res.

[42] Vasaikar SV, Straub P, Wang J, Zhang B (2018). LinkedOmics: analyzing multi-omics data within and across 32 cancer types. Nucleic Acids Res.

