# Supplementary figures and images for: KDELR2-KIF20A axis facilitates bladder cancer growth and metastasis by enhancing Golgi-mediated secretion
Source: Biol Proced Online. 2022 Sep 12;24:12. doi: 10.1186/s12575-022-00174-y (PMC9465899; doi:10.1186/s12575-022-00174-y)

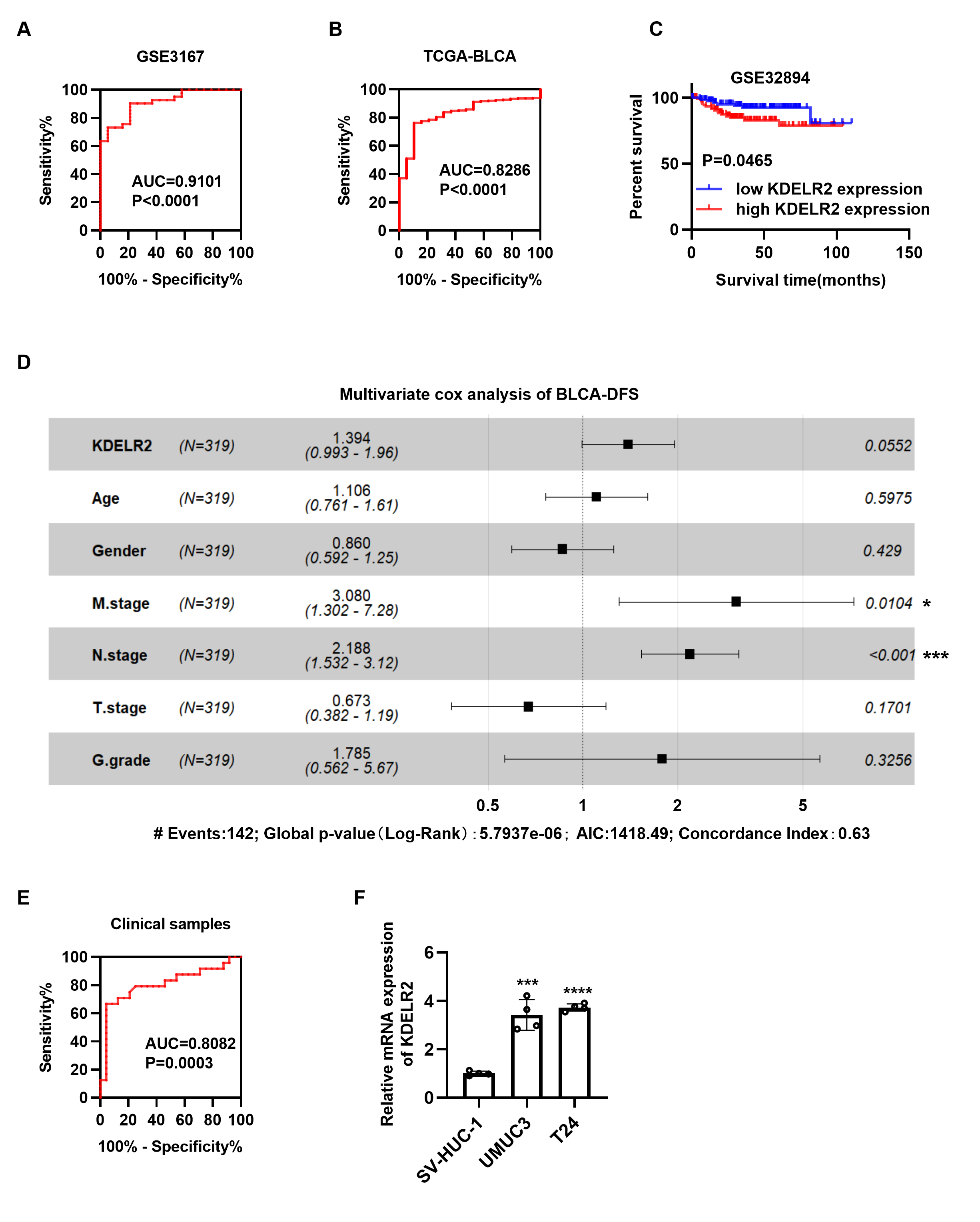

Supplement: Supplementary file 1 — Additional file 1: Figure S1. KDELR2 has a prognostic and diagnostic value in BCa. A-B Receiver operating characteristic (ROC) curves of KDELR2 in the GES3167 (normal, n = 19; tumor, n = 41) and TCGA-BLCA datasets (normal, n = 19; tumor, n = 408). (C) Kaplan–Meier curves of KDELR2 expression in patients with BCa in the GSE32894 dataset (high, n = 112; low, n = 112). (D) Multivariate analysis of KDELR2 mRNA level and DFS in patients with BCa. E ROC curves of KDELR2 in clinical samples (normal, n = 24; tumor, n = 24). F Detection of KDELR2 expression by qRT-PCR analysis in cell lines (n = 4). p < 0.001, ***; p <0.0001, ****. [file 12575_2022_174_MOESM1_ESM.tif]

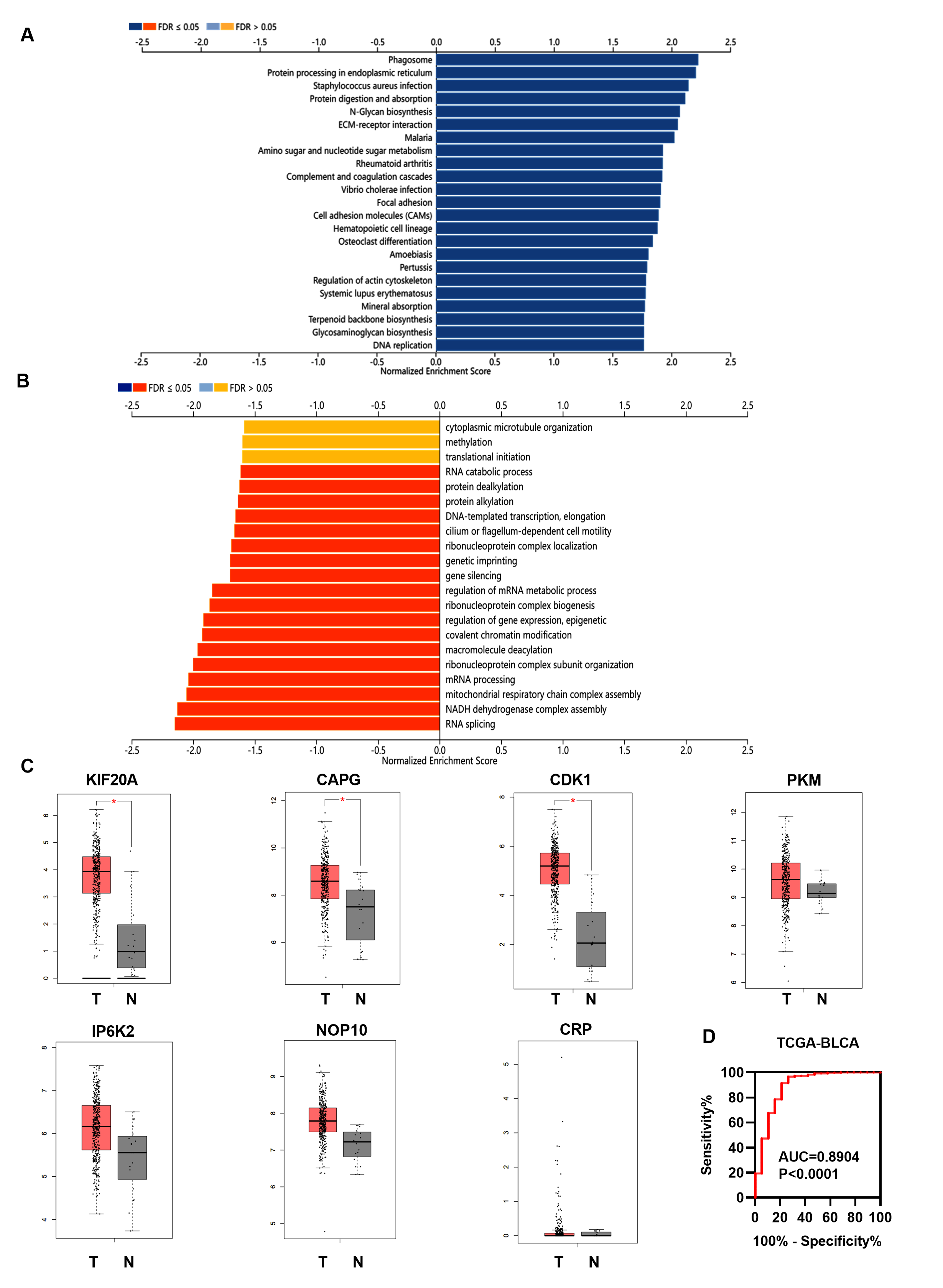

Supplement: Supplementary file 2 — Additional file 2: Figure S2. A-B Biological processes of KDELR2 mainly involved in BCa analysed by KEGG pathway enrichment and GO analysis with the LinkedOmics tool. C Expression profiles of KIF20A, CDK1, CAPG, NOP10, PKM, IP6K2, and CRP mRNA in the TCGA-BLCA datasets (normal, n = 19; tumor, n = 408). D ROC curves of KIF20A in TCGA-BLCA datasets (normal, n = 19; tumor, n = 408). p < 0.05, *. [file 12575_2022_174_MOESM2_ESM.tif]

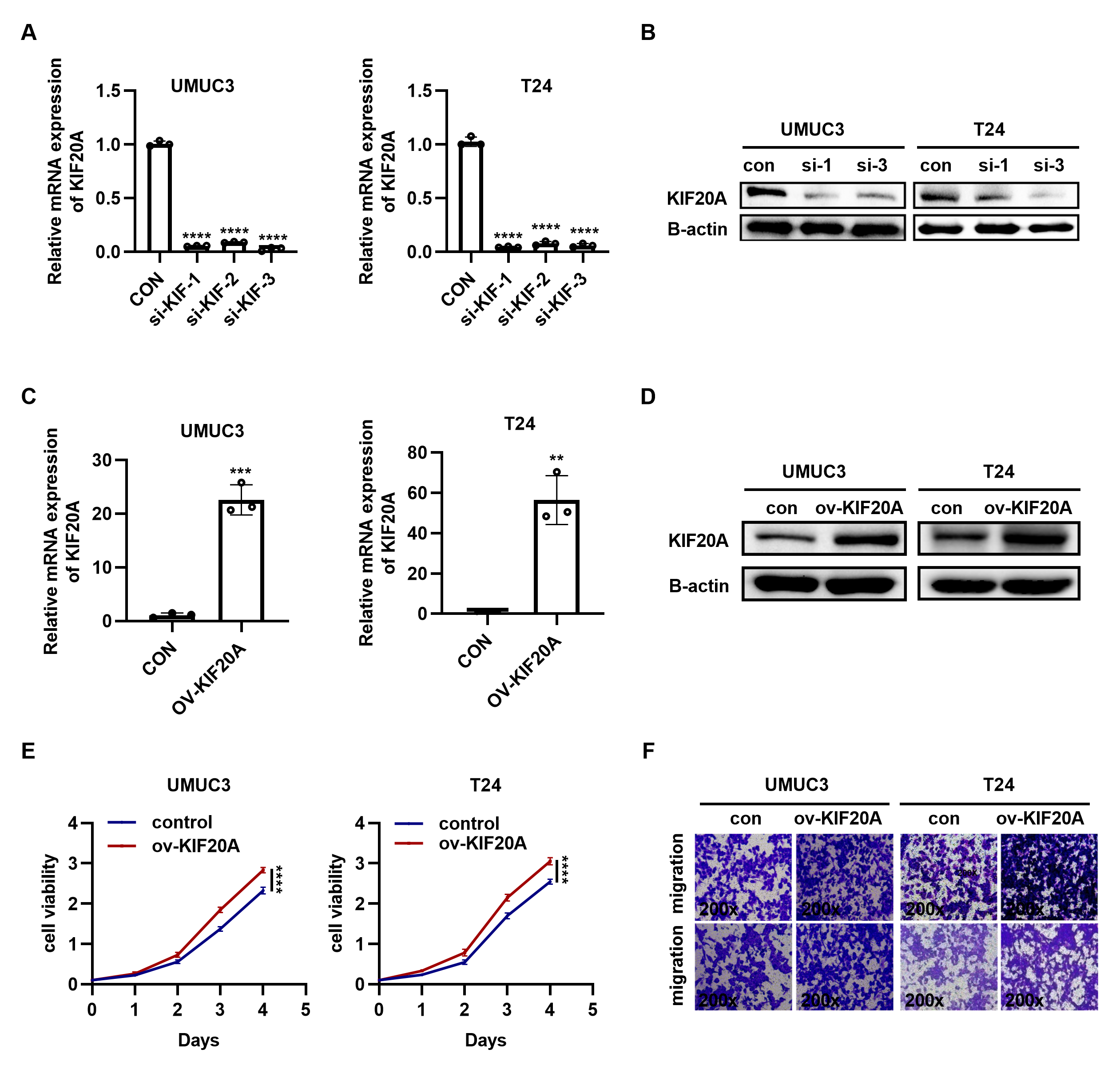

Supplement: Supplementary file 3 — Additional file 3: Figure S3. Identify the expression of KIF20A and its biological function. A-D Identification of knockdown or overexpression efficiency of KIF20A by qRT-PCR and western blot analysis (n = 3). E Proliferation analysis of UMUC3 or T24 cells in the control and KIF20A-overexpressing groups (n = 4). F Migration and invasion (200x) of BCa cells in the control and KIF20A-overexpressing groups (n = 3). p < 0.01, **; p < 0.001, ***; p <0.0001, ****. [file 12575_2022_174_MOESM3_ESM.tif]
